# On the Function of Boredom

**DOI:** 10.3390/bs3030459

**Published:** 2013-08-15

**Authors:** Shane W. Bench, Heather C. Lench

**Affiliations:** Department of Psychology, Texas A&M University, 4235 TAMU, College Station, TX 77843, USA; E-Mail: swbench@gmail.com

**Keywords:** boredom, emotion, functional accounts, negative emotion, discrete emotion

## Abstract

Boredom is frequently considered inconsequential and has received relatively little research attention. We argue that boredom has important implications for human functioning, based on emotion theory and empirical evidence. Specifically, we argue that boredom motivates pursuit of new goals when the previous goal is no longer beneficial. Exploring alternate goals and experiences allows the attainment of goals that might be missed if people fail to reengage. Similar to other discrete emotions, we propose that boredom has specific and unique impacts on behavior, cognition, experience and physiology. Consistent with a broader argument that boredom encourages the behavioral pursuit of alternative goals, we argue that, while bored, attention to the current task is reduced, the experience of boredom is negative and aversive, and that boredom increases autonomic arousal to ready the pursuit of alternatives. By motivating desire for change from the current state, boredom increases opportunities to attain social, cognitive, emotional and experiential stimulation that could have been missed. We review the limited extant literature to support these claims, and call for more experimental boredom research.

Either the well was very deep, or she fell very slowly, for she had plenty of time as she went down to look about her and to wonder what was going to happen next. First, she tried to look down and make out what she was coming to, but it was too dark to see anything; then she looked at the sides of the well, and noticed that they were filled with cupboards and book-shelves; here and there she saw maps and pictures hung upon pegs.

Lewis Carroll ([1], pp. 10–11)

## 1. Introduction

In the classic story *Alice’s Adventures in Wonderland*, Alice chases a white rabbit down a well and then begins a long fall. While falling, Alice’s attention shifts frequently. Alice begins by considering what is beneath her (the outcome of her fall). Next, she notices the cupboards surrounding her, and even interacts with them. She then considers how she will relate this fall to other everyday falls, and eventually simulates the conversations she will have with the people she meets on the other side of the Earth. Alice does not remain afraid and focused on the outcome of her fall (what is beneath her). While this example is fictional and caricatured, it reflects a truth about every day emotional experience—the intensity of all emotions fades over time and attention shifts to novel stimuli. What motivates people to seek out new goals as previous experiences fade? We propose that as the intensity of an emotional experience diminishes, the emotional state of boredom will encourage people to pursue alternate goals and experiences, including experiences likely to elicit negative emotions. 

The experience of boredom is ubiquitous and occurs frequently in daily life across a variety of cultures [2,3,4,5]. Although the experience of boredom is common, and has been a topic of scientific interest since the ancient Greeks [6], defining boredom has proven a difficult task, in part because it is not clear why people experience boredom. Researchers have defined boredom according to several different outcomes associated with boredom, including arousal [7], attention [2], meaning of the current situation [8,9,10,11], and cognition [12]. Perhaps the most widely used definition of boredom is “the aversive experience of wanting, but being unable, to engage in satisfying activity” ([2], p. 482). In modern psychology, boredom has been considered largely inconsequential for human functioning—a fleeting state that results from monotonous tasks or limited external stimulation [13]. However, the sheer frequency with which boredom is experienced across individuals and cultures suggests that the state may be important for human functioning. Further, a growing body of research suggests that boredom proneness (trait boredom) has important implications for a multitude of real world outcomes. Trait boredom has been linked to gambling [14], drug and alcohol abuse [15,16], binge eating [17], dropping out of school [18] and depression and anxiety [16,19] (see [2] and [20] for reviews). Although this work is based on findings regarding trait boredom (the tendency to experience boredom), and these findings have informed our proposal, it is important to note that trait and state boredom may be quite different [20], similar to other emotions in which trait and state emotions are qualitatively different [21,22]. We build on these findings to argue that boredom serves a functional purpose.

While applied and clinical research on trait boredom appears to be growing, relatively little experimental work has focused on the effects of state boredom. In fact, a recent meta-analysis revealed 510 articles that dealt with the impact of happiness, sadness, anxiety, or anger [23], approximately 128 articles per emotion, but a similar search revealed only 12 articles detailing experimental studies focused on the impact of boredom (see [23] for search criteria). The purpose of this paper was to propose a function of boredom through review of relevant research, and encourage further experimental work to support this claim. Our proposal is guided by work on the trait of boredom proneness and the relatively little experimental research that has focused on boredom; however, our intention was not to review the boredom literature, but to provide predictions to guide future empirical work. Specifically, we suggest that, although usually short-lived like other emotions, boredom is a commonly experienced functional emotional state that encourages the pursuit of alternative goals and experiences. We employ adaptive theories of emotion to integrate existent research on boredom and to propose new avenues for empirical work on boredom. 

## 2. Adaptive Theories of Emotion

Functional accounts of emotion argue that emotions arise from specific environmental conditions or problems, and serve to organize responses to those conditions [24,25,26,27,28]. Generally, emotions reflect the current status of progress toward a goal and give information about how well or poorly one is doing. Specifically, emotions indicate the status of goals, and cause changes in systems to enable goal directed behavior [29,30]. For instance, happiness is an indication of the success of a goal [23,31,32], anger signals a goal has been failed, but has a chance to be reinstated [23,33], sadness indicates goal failure with no hope for recovery [23,33,34], and anxiety is due to anticipated threat to important goals [23,33]. Thus, according to this perspective, each specific emotion signals that individuals need to take some action to attain goals or avoid negative outcomes. 

In addition to unique environmental precursors, these accounts typically posit that discrete emotions are associated with changes in behavior, cognition, experience, and physiology. For example, anxiety engenders behaviors intended to avoid threats, cognitions that highlight threats and coping, the subjective experience of anxiety and threat, and physiological changes that prepare the organism for self-preservation. A recent meta-analysis supported the general claim that discrete emotions (*i.e.*, happiness, sadness, anger, anxiety) are correlated with changes across these outcomes [23]. 

Although each emotion (happiness, sadness, anger, and anxiety) signals that action is needed to accomplish goals in a functional account of emotion, these models do not indicate when or why people will disengage from current goal pursuits. The models include the possibility that extreme sadness, indicating that a goal is irrevocably lost (e.g., a loved one has died), might result in goal switching. Other than this, though, it is not clear when or why people will take up new goals, which, according to qualitative evidence, people do frequently [35,36]. We propose that, as the intensity of emotional responses fades during goal pursuit, boredom acts as an emotional signal that current goal pursuits should be abandoned and that new goals should be actively selected and pursued.

## 3. Boredom as an Emotion

Previous theoretical work has proposed that boredom is a discrete emotion [8,9,10,11,20,37,38,39,40,41]. Building on this previous work, we rely on an adaptive theory of emotion to propose that perception of specific situational factors will result in the experience of boredom and associated responses [30,32,42,43]. Specifically, we argue that boredom arises from the perception that the current situation is no longer stimulating, as reflected in diminishing emotional response to the situation. Boredom then organizes responses to this situation that encourage people to seek alternative goals and experiences, even if those experiences might result in negative emotions. Functional accounts of emotion hold that emotions indicate the status of goals [29,30], and, similarly, we argue that boredom reflects the status of goals. We propose that the goal associated with boredom is the importance of persistence toward a current goal. Working toward a goal elicits emotional states. As this affective experience begins to weaken, the benefit of persistence towards the goal also reduces, as one is no longer succeeding or failing at the same rate, and as a result, an alternative goal has the potential for greater reward. Boredom is the emotional indication to pursue an alternative goal. Indeed, recent theoretical and experimental work have proposed that boredom facilitates pursuit of activities that increase the perception of meaning [8,9,10,11], and this is consistent with the idea that boredom facilitates the pursuit of alternative goals when current goals are not fulfilling. 

It is well known that emotions fade over time [44,45,46] (see [47] for a review), becoming less intense. Once a goal is accomplished (happiness), blocked (anger), threatened (anxiety), or lost (sadness), and has been in such a state long enough for the emotional response to begin to fade, we suggest that the emotional system signals that it is time to move on to other pursuits. Boredom, we propose, is that signal. Boredom would occur as intense or weak reactions fade. The time required for boredom to ensue would be determined by the duration of a reaction. Consider, for example, the experience of happiness after a goal is attained [31]. Depending on how important the goal was, one might spend a short time (e.g., after being gifted a balloon) or a long time (e.g., after marrying a soul mate) basking in the happiness created by accomplishing that goal. But even emotional responses to important events are short-lived [24], and affective intensity reduces with time and exposure [44], so the state of happiness would not persist indefinitely. After a honeymoon, eventually the intensity of happiness would fade, boredom with lounging around and communicating with one person would begin to intrude, and other goal pursuits would be taken up (e.g., returning to work). Even the experience of intense fear caused by a potential threat to life could fade as the threat persists, shifting attention to other concerns. Consider, for example, a skydiver that initially feels mind-numbing fear as they begin to fall. As the fall persists, the skydiver’s experience may become similar to Alice’s fall down the well - they begin to notice their surroundings, perhaps the beautiful view and perspective that this position affords. We do not argue that one would necessarily experience boredom in this situation, but rather believe this common experience illustrates how attention can shift as emotional intensity begins to fade, even for very intense emotion. Because emotions are part and parcel of effective goal pursuit, there would be no motivation to pursue new goals if emotions did not fade over time, allowing for disengagement from that goal. That is, an emotional state is only functional if it ceases to persist. Always being happy, angry, sad or afraid about the same goal would have little adaptive value. As the intensity of these (and other) emotions begins to subside, boredom arises to indicate a new goal should be pursued and motivate responses to switch goals. 

Importantly, boredom does not discriminate the valence of a goal that should be switched to, it simply encourages changing to a new goal. We propose that, because boredom motivates a desire for change, the specific goal that is desired would be dependent upon the current state. That is, reference points would determine the goal that boredom motivates, as the desirable goal would be one that is different from the current situation. Due to this, boredom could encourage changes that result in negative emotion, such as looking attentively at an animal corpse on the side of road during a long drive. Boredom could even motivate goals that result in actual risk, such as high stakes gambling or sex with multiple partners. While pursuing negative outcomes and emotions (ant-hedonic behaviors) is in many ways maladaptive, there may be some benefits as well. Anti-hedonism permits the attainment of opportunities that would have otherwise been missed [48]. For example, gambling provides a high probability of loss, but can also provide fast and easy gains. Without gambling there is no chance of experiencing the probable loss, but there is also no chance of attaining the gain. Similarly, unprotected sex with multiple partners increases the chance of sexually transmitted infection, but it also increases the chance of conception and reproduction. There may also be benefit to exploration. If a violent river has never been crossed due to extreme danger, there is no way of knowing what potential gains are available on the other side of the river. These situations entail probable negative emotions, but also the chance for gains and for information about the environment. Anti-hedonism allows for the attainment of possible gains that would otherwise be overlooked. This proposed function is based on an adaptive account of emotion; future research is needed to investigate these claims. 

The function that we propose for boredom is somewhat similar to that proposed for other negative emotions that encourage goal pursuit, most notably anger and frustration [29]. We argue that boredom is motivating and encourages action towards a new goal as emotional intensity fades. Anger and frustration have been proposed to also motivate action to attain goals, particularly when one is relatively close to goal attainment. However, the environmental conditions that result in boredom and anger are actually quite different, and the responses to those conditions are also different. Anger results when an identified object or person is blocking a specific recognized goal, but a chance to attain the goal remains [49,50]. For example, a person might be angry if while rushing to a meeting they are slowed by unexpected traffic. In this example the goal would be making it to the meeting on time, and the object blocking the goal would be the traffic. Boredom, in contrast, does not require a clearly identifiable goal (beyond change from the current state), and there is not a recognizable object blocking that goal. For instance, while waiting in slow moving traffic for an extended amount of time, a person might look attentively at a gruesome accident as they eventually pass. In this example, the goal would be a change from the current experience (*i.e.*, waiting in slow moving traffic) and an available alternative experience would be the accident. To take the example further, a person might initially be angry that they are slowed in traffic, as their goal is to reach their destination, and it is being blocked by the traffic. With time, however, the intensity of the anger experience would begin to fade, and the person would begin to become bored. Boredom would then motivate pursuing an alternative goal, such as observing the damage of a car accident. Thus, we propose that boredom arises as emotional intensity fades and one approaches a “neutral” state. 

It is also important to distinguish two affective states involving dissatisfaction with the current situation: boredom and apathy. Apathy results from recognition of complete failure or helplessness [29] and is characterized by a lack of motivation [51] and a failure to seek alternatives. In contrast, we propose that boredom results from recognition that the current goal is no longer stimulating (*i.e.*, is associated with less intense emotion) and is characterized by motivation to change the current situation and seek alternatives [52]. Increased motivation would allow for the pursuit of alternate goals. For example, if a person was unsatisfied with their current romantic relationship, but is not attempting to improve their relationship or seek alternatives because they view the situation as hopeless, it would be apathy. A person bored with their current romantic relationship would likely seek to change the situation, either by pursuing new goals in their current relationship or seeking an alternative partner. The difference in resulting motivation is a crucial distinction between boredom and apathy, as both states are sometimes colloquially referred to as “boredom”, but they have very different effects. Indeed, recent research has demonstrated that apathy and boredom are discrete constructs [53].

Based on our proposal regarding the environmental conditions that give rise to boredom, we next discuss the potential specific and unique impacts of boredom on cognition, behavior, experience, and physiology. These impacts are outlined in Table 1. Akin to other discrete emotions, the impacts of boredom should help resolve the conditions that elicit boredom [23]. Specifically, boredom should encourage the pursuit of goals and experiences that differ from those currently experienced. In many cases, this would come in the form of novel stimulation which would introduce opportunities for cognitive and social growth, even if the alternative situations might elicit negative emotion. That is, by creating a desire for change, boredom encourages people to alter their current situation, which permits the attainment of opportunities that might have been missed. 

**Table 1:** Proposed effects of boredom and the manifestation of those effects.

	Effect	Manifestation
Behavior	Motivational—Pursue a new goal Social—encourage others to create new situation	Preferences for novel stimuli; preference for risk. Express boredom through facial movement and nonverbal cues such as eye rolling.
Cognition	Motivational—pursue a new goal	Attention devoted to novel stimuli; mind wandering.
Experience	Motivational—pursue a new goal	Experience the current state as negative and averse; elicits avoidance of a state of boredom.
Physiology	Motivational—pursue a new goal	Increased autonomic arousal.

## 4. Behavior

The proposed function of boredom to motivate pursuit of alternative goals and experiences, even those that might elicit negative emotions, should be reflected in behavior. Research examining the relationship between trait boredom and risk taking behaviors partially supports this proposal, although trait boredom may well differ from the effects of state boredom [20]. A trait tendency to experience boredom has been associated with several risk taking behaviors, including drug and alcohol abuse [15,16], binge eating [17], dropping out of school [18] and problem gambling [14,54]. Although risky behavior may elicit negative emotions, it represents an opportunity to pursue alternative goals likely to stimulate and elicit intense emotional responses [48], and thus, these findings, though based on trait boredom, are consistent with our proposal. 

The impact of the experience of state boredom on behavior has received relatively little research attention. In one of the few studies we are aware of, participants completed a task to induce boredom, and then were told that, due to time constraints, they would only be able to complete one of two follow up studies. Participants were then allowed to choose between a study described as completing questionnaires of common events (e.g., how often do you eat breakfast) or a study involving watching a film that would elicit intense negative emotions and high arousal and then answering questions. A small portion of participants chose to complete the study described as intensely negative and arousing. Participants that chose the negative arousing follow up study were those that experienced a lower level of sensory stimulation [55]. Based on these findings, and building on previous theory [7,48], we speculate that boredom should motivate preferences for novel stimuli, including risky situations. This preference should be reflected in choices of unfamiliar situations and objects (e.g., interacting with strangers, choosing challenging tasks). Preference could be given to experiences that are likely to elicit positive emotions, such as a funny movie, or negative emotions, such as a sad movie. Choosing situations likely to elicit negative emotions, such as watching a sad movie or horror film, may be particularly likely after boredom was elicited by recent experience with a situation that elicited happiness (that has now faded in intensity). That is, the new outcome that is pursued would likely be determined by the situation that has now faded and produced boredom; if a person became bored during a positive experience (e.g., a funny movie), a negative experience might become desired and pursued (e.g., a sad movie). Thus, boredom could motivate behavior to pursue novel opportunities, either positive or negative.

Preferences for novelty following boredom could also be reflected in changes in arousal. With repeated exposure, events become familiar and the amount of arousal they produce diminishes [7]. Boredom that results from acclimatizing to a low arousal situation (e.g., reading a paper) might encourage pursuit of a high arousal activity (e.g., an intense workout at the gym). Similarly, recent experience with a high arousal activity (e.g., speeding through traffic) might encourage pursuit of a low arousal activity (e.g., listening to a lecture), as this activity represents a change in the level of arousal. Interestingly, there may be individual differences in the tendency to prefer changes in arousal *versus* continuing high arousal. Some individuals, notably those high in sensation seeking, may prefer to seek additional arousing activities when their current arousal fades (*i.e.*, sensation seekers; [7,56]). A substance abuser that uses an ever increasing amount of a substance to attain a similar level of arousal would be an example of this type of pursuit. 

Another line of experimental studies investigating the impact of boredom on behavior has examined the role of meaning. The pursuit of meaning is proposed to be a fundamental aspect of human life, and provides a sense of purpose [57]. According to this perspective, boredom results from a meaningless situation [8], and engenders an urge to seek more meaningful activities. In a series of studies participants have sought out more meaningful outcomes after a boredom induction [9,10,11]. Specifically, while experiencing a state of high compared to low boredom, participants were more favorable of their in group, and treated out group members more harshly, as group membership provides a sense of meaning [9]. Further, when bored, participants were more likely to use nostalgia to return a sense of meaning to their lives, suggesting that memory was altered to promote a sense of meaning [11]. These important findings demonstrate that boredom motivates behavior to change the current situation. While these studies have focused on the importance of meaning in boredom, and have demonstrated the value of meaning, we contend that behavior is not always driven by meaning [58,59]. That is, boredom may sometimes motivate behaviors meant to increase a sense of meaning; however, more broadly speaking, boredom motivates behaviors with the intent to change the current situation. Other emotions can also, at times, motivate behaviors without meaning. For instance, a person might become anxious by a passing shadow. This would still result in an experience of anxiety, but there would not be an actual threat. Thus, boredom, like other emotions, could motivate behaviors without meaning. Importantly, we propose that boredom results from diminishing emotional stimulation in a situation and prompts responses to facilitate the pursuit of alternative goals.

Boredom may also serve a function in social situations, as emotions are proposed to have powerful impacts on interactions [26]. Typically the social function of emotion is fulfilled through communicating emotion to others through expression [26,28,42]. For instance, an expression of fear communicates that there is a present threat to others [28]. We propose that boredom expresses to others that a person is seeking change and stimulation, potentially prompting others to respond by assisting in this pursuit. Notably, these are expressions of boredom based on the proposed function of boredom, but there is limited empirical work related to the experience of boredom. The expression of boredom would be that of closed lips without flexion of the zygomatic muscle, this could serve as a demonstration of disengagement from the situation and others [60]. A recent study also found that the buccinators muscle is activated during experiences of boredom, differing boredom from shame and sadness [61]. Eyes should be open, but with the lids slightly drooped [61]. Gaze should not be directed toward anything specific. This would demonstrate to others that interest is lost, and that attention is not on them [60]. The eyes might also be rolled as a further demonstration that boredom is being experienced. The posture of boredom is slouched and hunched over [60,62], likely because no new goal has been identified. Overall, the expression of boredom has received little research attention. The extant research agrees with our proposed function of boredom, but much of this is based on a single study. Additional research is required to reliably establish expressions associated with boredom.

## 5. Cognition

The pursuit of alternative goals and experiences engendered by boredom is likely to be reflected in shifting attention to novel stimuli. This could be reflected in alternative external or internal stimuli. External stimuli would be any change in the environment. This could be moving to a different location, pursuing a new activity or challenge (e.g., quitting work to return to school), or engaging a different person or group of people. Internal stimuli could be changes in thoughts, affect, arousal, or attention (e.g., to stop attending to what is being read and start focusing on the birds heard outside the window). All of these changes would introduce new stimulation. 

Consistent with our proposal, boredom has powerful effects on attention (for a complete review see [2]). In particular, boredom makes it difficult to attend to a task that is currently being completed. Indeed, at the trait level, there is a relationship between boredom proneness and attention deficit hyperactivity disorder [63,64]. This relationship also holds in experimental work on state boredom. In multiple studies meant to assess the effect of boredom on vigilance, participants are required to monitor carefully for the appearance of certain stimuli. These stimuli are rarely presented, and the tasks are usually quite long (about 90 minutes). As the task progresses, reported level of boredom increases and task performance decreases. Participants also report that it is increasingly difficult to maintain attention [65,66,67]. One limitation of these studies is that boredom is manipulated by the same task on which performance is measured, meaning that it is unclear if boredom elicits a general tendency to limit attention to tasks or a general lack of attention to any task.

Consistent with our proposal that boredom shifts attention to alternative goals and experiences, research also suggests that boredom is associated with greater mind wandering. “Mind wandering” refers to shifts in attention away from a current task and toward unrelated cognitions and feelings [68,69]. Mind wandering to information unrelated to current tasks has been associated with a failure to engage attention to the current task [68], and may be caused by boredom. Recent research has suggested that mind wandering occurs frequently, and people feel more negative affect while mind wandering [3]. This might be due to people being more likely to mind wander to something positive than something negative [3]. Mind wandering to something more positive than the current situation could be an indication the current situation is of low functional importance; this would reduce satisfaction with the current task [70] and encourage change. When the mind wanders to a more negative event, satisfaction with the current activity may be bolstered, and encourage one to maintain attention. A recent theory of boredom suggests that an inability to sustain attention, and mind wandering, lead to boredom [2]. We contend, however, that mind wandering may be result from boredom. Specifically, as the intensity of an experience fades, the experience of boredom begins. Mind wandering is then motivated by boredom and is an attempt to find a new goal. It is also possible, however, that as an emotional experience begins to fade, the mind starts to wander as the increased attention associated with an emotional experience also fades, before a state of boredom is actually experienced. Extant research demonstrates that mind wandering is a negative experience [3,71], and that boredom and mind wandering are related [2], the causal relationship of the two, however, is currently unclear.

Finally, it has been suggested that boredom can increase creativity [72,73,74], despite the fact that folk ideas often consider boredom and creativity to be opposites [73,74]. In support of this claim, one study found that, when asked about the subjective positive outcomes of boredom, some participants listed increased creativity [72]. Future research should examine the effects of an induced state of boredom on creativity tasks. 

## 6. Experience

The experience of boredom should be negative and aversive, creating a desire to change from the current state and avoid future states of boredom. Consistent with this proposal, research has found that boredom is reported to be a negative affective experience [10,63,75,76,77,78]. The experience of boredom as a negative state appears to arise in part from a subjective sense of time passing slowly [79]. In one study, participants completed a task in front of a clock. In one condition, the clock showed that 10 minutes had passed and in the other condition it showed 30 minutes had passed. In both conditions the actual time of the experiment was 20 minutes (*i.e.*, the clocks were manipulated to show more time or less time had passed than actually had). Participants reported more boredom when the clock showed they had worked on the task for 10 minutes, compared to 30 minutes. This is due to participants feeling like more time had passed (and it actually had), causing them to report more boredom, as boredom could account for time seemingly passing slowly [80]. Boredom also includes a sense of being unable to escape an undesirable moment [2,12]. This would create a feeling of being trapped in the current experience, and we propose would encourage attempts to change the experience. The slow perception of time passage, and feeling incapable of escaping, likely make the experience of boredom subjectively more negative and increase the desire to seek alternative goals and experiences.

A recent series of studies assessed the self-reported experience of boredom compared to other emotions [10]. The self-reported feelings, thoughts, action tendencies, actions, and motivational goals experienced while bored were distinct from those of anger, frustration and sadness. Specifically, boredom was reported to uniquely include simultaneous feelings of restlessness and a lack of challenge. Distinct thoughts included that the current situations served no purpose. Participants also reported a desire for doing something completely different and a desire to do something with purpose [10]. These findings provide important differences between the experience of boredom and other similar emotions and, although differences among emotions were self-reported, suggest directions for future work to establish the effects of boredom.

## 7. Physiology

Boredom should be associated with increased autonomic arousal to enable the pursuit of alternative goals and experiences, but potential physiological changes due to boredom have received little research attention. In two studies boredom was manipulated through the use of a vigilance task, or a repetitive writing task (*i.e.*, writing the letters “cd” repeatedly), or participants wrote stories to describe the events in a picture (the second study was a within-subjects design, and counterbalanced the order of condition). While completing the task, participants galvanic skin potential (study 1), skin conductance and heart rate were recorded (study 2). Results showed that higher levels of reported boredom were accompanied by increased autonomic arousal (*i.e.*, heart rate and galvanic skin response; [81]). This finding might seem counterintuitive, based on the conventional lethargic conceptualization of boredom. Indeed, the extant research on the physiological effects of boredom is mixed, with some findings that boredom is associated with low arousal [82], some findings that boredom is a high arousal state [72,81], and some findings that boredom can be associated with both low and high arousal [2,6,20]. These mixed findings appear to result from a failure to distinguish between apathy (a low arousal state) and boredom (potentially a high arousal state). We contend that, because boredom encourages seeking alternative goals and experiences, it should be associated with increased activity from the autonomic nervous system. In addition to the experimental findings above, this claim is indirectly supported by findings that under-stimulated (*i.e.*, bored) rats run faster [83], and children exposed to a stimulus for a long period of time respond faster to a signal noise than children exposed for a short period [84]. These findings suggest that boredom prepares for action, likely due to increased autonomic arousal. 

## 8. Conclusions

Boredom is frequently and commonly experienced, suggesting that it plays a valuable role in human goal pursuit. We propose that boredom is a discrete functional emotion, and serves to encourage people to seek new goals and experiences. Boredom provides a valuable adaptive function by signaling it is time to pursue a new goal. Much like Alice becoming distracted from her fear of falling and shifting her attention towards the cupboards and her upcoming conversations; we propose that boredom will motivate the pursuit of new goals as the intensity of the current experience fades. Experimental research is needed to test these claims, but the current paper provides a much needed direction for basic boredom research. 
